# Using DNA testing for the precise, definite, and low-cost diagnosis of sickle cell disease and other Haemoglobinopathies: findings from Tanzania

**DOI:** 10.1186/s12864-021-08220-x

**Published:** 2021-12-16

**Authors:** Heavenlight Christopher, Adam Burns, Emmanuel Josephat, Julie Makani, Anna Schuh, Siana Nkya

**Affiliations:** 1 Sickle cell programme, Department of haematology and Blood Transfusion, Muhimbili University of Health and Allied Sciences, Dar es Salaam, Tanzania; 2 Oxford Molecular Diagnostics Centre, University of Oxford, Oxford, UK; 3 Department of Biological Sciences, Dar es Salaam University College of Education (DUCE), Dar es Salaam, Tanzania

**Keywords:** Comprehensive care, Newborn screening, Sickle cell disease, Haemoglobinopathies, Nanopore, DNA sequencing

## Abstract

**Background:**

Sickle cell disease (SCD) is an important cause of under-five mortality. Tanzania is the 5th country in the world with the highest births prevalence of SCD individuals. Significant advances in the neonatal diagnosis of SCD using rapid point-of-care testing have been made. However genetic confirmation is still required for positive cases, in uncertain cases, in multiply transfused patients, to resolve compound heterozygosity (Hb S/ β^0^ Thal or Hb S/ β^+^ thal) not uncommon in the coastal regions of East Africa and increasingly also for pre-marital counselling and potentially for future curative approaches such as gene therapy. The currently available DNA tests are prohibitively expensive. Here, we describe an easy-to-use, affordable and accurate β-globin sequencing approach that can be easily integrated within existing NBS for SCD and other haemoglobinopathies especially in Low- and Middle-income Countries.

**Aim:**

To evaluate an affordable DNA technology for the diagnosis of Sickle cell disease and other haemoglobinopathies in a resource-limited setting.

**Methods:**

Laboratory-based validation study was conducted by Muhimbili University of Health and Allied Sciences and the University of Oxford involving sequencing of the entire β -haemoglobin locus using the Oxford Nanopore MinION platform. A total number of 36 Dried blood spots and whole blood samples were subjected to conventional protein-based methods (isoelectric focusing, HPLC), and/or sequenced by the Sanger method as comparators.

**Results:**

Sequencing results for SCD using the MinION were 100% concordant with those from the Sanger method. In addition, the long-read DNA sequencing method enabled the resolution of cases with unusual phenotypes which make up 1% of all children in Tanzania. The cost is £11/ sample for consumables, which is cheaper compared to other sequencing platforms.

**Conclusions:**

This is the first report of a comprehensive single DNA assay as a definitive diagnostic test for SCD and other haemoglobinopathies. The test is fast, precise, accurate and affordable.

## Introduction

Sickle cell disease (SCD) refers to a group of inherited red blood cell disorders that occur due to mutations in β-globin genes, one of which is invariably haemoglobin S (HbS), a variant produced as a result of glutamic acid to valine substitution at position 6 of the beta-globin chain, the underlying mutation being HBB: c.20A > T. Deoxy-HbS is prone to polymerization, disrupting red blood cell shape, function, and life span. HbS can be co-inherited with β-thalassaemia and other haemoglobin variants that might impact clinical phenotypes. SCD is generally characterized by chronic haemolytic anaemia and recurrent vaso-occlusions, leading to painful crises, multi-organ impairment, and sometimes premature death [1, 2].

Worldwide it is estimated that > 300,000 babies are born annually with SCD and that these numbers will likely increase to 404,200 in 2050 [3]. Most of these babies are born in sub-Saharan Africa and India [4–6], where SCD contributes significantly to early childhood mortality and morbidity [7, 8]. In 2006, the WHO, at its 59th health assembly, declared SCD as a significant public health burden in Africa [9, 10].

Currently, the diagnosis of SCD and other haemoglobinopathies relies on protein-based assays with isoelectric focusing (IEF) as the first-line sickle cell screening test, followed by confirmation of positive cases by high-performance liquid chromatography (HPLC) as a second-line screening method. Although these are standard methods, they require transport of the sample to a central laboratory, expensive equipment, experienced operating personnel and genetic confirmation of positive cases. Point-of-care (POC) immunoassay methods, such as Sickle SCAN and HemoTypeSC, detect the altered sickle epitope of the beta chain and have greatly facilitated neonatal screening through rapid identification of likely affected babies in high-prevalence regions from dried blood spots (DBS) from a heel prick performed shortly after birth. However, they only provide a qualitative result of the presence or absence of Hb A, S, and C, cannot diagnose other haemoglobinopathies [11–16] and require confirmatory testing.

The capacity for genetic testing for SCD and other haemoglobinopathies in Africa is largely limited. In addition to confirmation of positive cases, it is also important to (1) resolve uncertain or complex cases that make up about 1% of cases overall, for example, multiply transfused patients or compound heterozygous individuals (Hb S/ β^0^ Thal or Hb S/ β^+^ thal), (2) inform pre-marital counselling and (3) identify suitable candidates for curative approaches such as gene therapy. However, conventional genetic testing using different PCR approaches and Sanger sequencing is labour intensive and further adds to the complexity and cost of current protein-based laboratory methods.

In the current study, we, therefore, focused on developing an assay and evaluating the use of nanopore sequencing technology as an affordable, low-maintenance genetics test. Single-molecule, or third-generation, sequencing, such as that offered by the MinION platform (Oxford Nanopore Technologies, Oxford, UK) enables the sequencing of DNA of long linear read lengths (1-100kbp). This capability, in combination with the rapid sequencing times, the low initial capital cost required for equipment and maintenance, makes the MinION an attractive DNA sequencing device for the genetic characterisation of SCD and beta-haemoglobinopathies that will greatly facilitate future research efforts based in low- and middle-income-countries [17].

## Materials and methods

### Study design and area

This was a laboratory-based validation study involving newborns and adults at Muhimbili National Hospital (MNH) and Temeke Regional Hospital, Dar es Salaam, Tanzania. MNH is the national tertiary health facility and has been offering SCD services, both clinical and laboratory, for the past three decades. Temeke Regional Hospital, is a referral hospital for the Temeke municipality in Dar es Salaam, Tanzania, which has recently established SCD services. Both MNH and Temeke Regional Hospital each provide services for residents in Dar es Salaam and the neighbouring regions.

### Study population

This study involved 18 dB samples from newborns screened by conventional testing with IEF and/or HPLC and were found to be either homozygous or heterozygous for sickle cell disease or had an unidentified haemoglobin band/ variant by IEF and HPLC. In addition to recruiting newborns from the SCD screening programme, we identified 18 patients from the adult haematology clinics at MNH, Temeke and archived buffy coat from previous SCD study: 2 had sickle trait (Hb AS) but described painful crisis and anaemia, 4 were members of the same family with a history of anaemia and suspected thalassemia and 12 with confirmed HbS/β^+^ thalassemia. Demographic and contact details were collected before the collection of the blood sample using a standard proforma and these were later entered into a project database developed in MySQL (Sun Microsystems Inc., Santa Clara, CA, USA). Purposive sampling was used to select individuals who participated in this study.

**Fig. 1:**
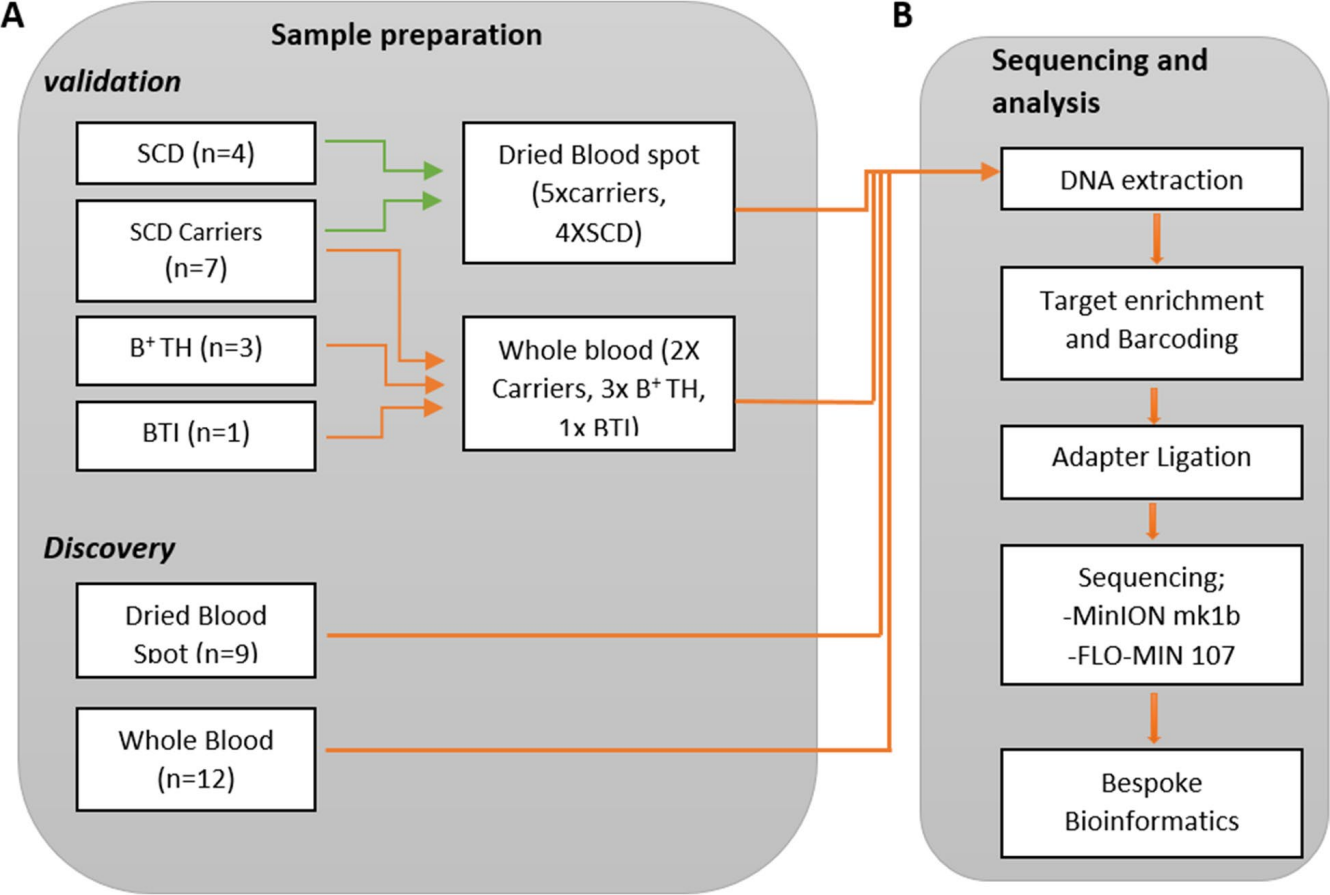
Details of the experimental methods used for both validation and discovery cohorts. **A** The validation cohort was selected for a mixture of confirmed clinical diagnoses and both WB and DBS sources. The discovery cohort comprised 9 dB and 12 WB samples. **B** Samples were prepared for sequencing according to DNA source and sequenced on a MinION mk1b instrument. The resulting sequencing reads were processed locally using a pipeline designed to identify constitutional variants in the β-globin gene. SCH = Sickle Cell Heterozygote, B + TH = Beta Thalassaemia Heterozygote, BTI = Beta Thalassaemia Intermedia, SCD = Sickle Cell Disease

To test the performance of the assay on DNA from both whole-blood and DBS samples, we grouped our samples into two validation cohorts and a discovery cohort (Fig. 1a). The first validation cohort comprised the 6 individuals from the adult haematology clinics, with DNA extracted from whole blood samples. The second validation cohort consisted of 9 newborn samples, with DBS derived DNA. All samples in the validation cohorts had been previously screened by IEF and HPLC (Table 1). The discovery cohort consisted of an additional 6 newborn DBS and 12 adult whole-blood samples (Table 2).

**Table 1:** Patient characteristics of the validation cohort. Blood samples are not routinely taken for FBC or HPLC as part of newborn SCD screening. ‘- ‘= data not collected

Sample ID	Sample Type	Age	Sex	Hb (g/dl)	MCV (fl)	Hb S	Hb A	Hb F	Hb A2	Diagnosis from standard test (genetic or IEF)	Concordance with Nanopore sequencing
FH	WB	21	F	12.4	78.8	36.3	55.8	3.3	3.3	Sickle cell heterozygote	yes
G213707J	WB	30	F	10.9	51.4	–	80.9	1.1	7.8	Beta+ Thal heterozygote	yes
G213708H	WB	33	M	11.4	47.2	–	81.3	1.2	6.7	Beta+ Thal heterozygote	yes
G213709R	WB	5	F	7.9	47.3	–	1.7	97.9	3.8	Beta Thal intermedia	yes
G213711L	WB	–	F	10.5	47.0	–	80.7	9.8	6.5	Beta+ Thal heterozygote	yes
Z	WB	5	F	8.5	74.8	38.2	51.2	5.6	3.6	Sickle cell heterozygote	yes
TMK 1196	DBS	Newborn	–	–	–	14.2	0.0	74.8	1.6	Sickle cell disease	yes
TMK 1258	DBS	Newborn	–	–	–	5.3	6.6	82.0	–	Sickle cell heterozygote	yes
TMK 1259	DBS	Newborn	–	–	–	4.8	7.4	80.6	–	Sickle cell heterozygote	yes
TMK 1260	DBS	Newborn	–	–	–	8.1	13.0	69.1	0.5	Sickle cell heterozygote	yes
TMK 1267	DBS	Newborn	–	–	–	3.8	4.6	83.5	1.3	Sickle cell heterozygote	yes
TMK 1276	DBS	Newborn	–	–	–	9.5	16.8	62.7	0.8	Sickle cell heterozygote	yes
TMK 1287	DBS	Newborn	–	–	–	12.4	0.0	80.6	0.5	Sickle cell disease	yes
TMK 1345	DBS	Newborn	–	–	–	12.2	0.0	78.9	1.4	Sickle cell disease	yes
TMK 1536	DBS	Newborn	–	–	–	–	–	–	–	Sickle cell disease	yes

**Table 2:** Summary of all non-synonymous variants obtained through conventional screening methods, confirmatory Sanger Sequencing, and nanopore sequencing

Sample ID	Sample Type	Cohort	Results from conventional screening		Results of nanopore sequencing				Final Diagnosis
			Method	Result	Mutation	Read Depth (x)	VAF	Het/Hom	
FH	WB	Val	Sanger	Glu7Val (Het)	Glu7Val	1645	0.46	Het	Sickle cell heterozygote
G213707J	WB	Val	Sanger	Met1Thr (Het)	Met1Thr	49,715	0.56	Het	Beta^+^ thal heterozygote
G213708H	WB	Val	Sanger	Met1Thr (Het)	Met1Thr	43,165	0.44	Het	Beta+ thal heterozygote
G213709R	WB	Val	Sanger	Met1Thr (Hom)	Met1Thr	80,434	0.77	Hom	Beta+ thal intermedia
G213711L	WB	Val	Sanger	Met1Thr (Het)	Met1Thr	127,405	0.41	Het	Beta+ thal heterozygote
Z	WB	Val	Sanger	Glu7Val (Het)	Glu7Val	4744	0.50	Het	Sickle cell heterozygote
TMK-1196	DBS	Val	IEF	FS	Glu7Val	91,193	0.81	Hom	Sickle cell disease
TMK-1258	DBS	Val	IEF	FAS	Glu7Val	118,680	0.45	Het	Sickle cell heterozygote
TMK-1259	DBS	Val	IEF	FAS	Glu7Val	46,389	0.55	Het	Sickle cell heterozygote
TMK-1260	DBS	Val	IEF	FAS	Glu7Val	32,431	0.55	Het	Sickle cell heterozygote
TMK-1267	DBS	Val	IEF	FAS	Glu7Val	38,726	0.55	Het	Sickle cell heterozygote
TMK-1276	DBS	Val	IEF	FAS	Glu7Val	41,793	0.53	Het	Sickle cell heterozygote
TMK-1287	DBS	Val	IEF	FS	Glu7Val	86,884	0.95	Hom	Sickle cell disease
TMK-1345	DBS	Val	IEF	FS	Glu7Val	17,534	0.85	Hom	Sickle cell disease
TMK-1536	DBS	Val	IEF	FS	Glu7Val	33,070	0.93	Hom	Sickle cell disease
Sample 144	WB	Disc	Sanger	Beta+ Thal	c.92 + 1G > A	5849	0.44	Het	Beta+ Thal, Sickle cell heterozygote
					Glu7Val	5845	0.48	Het	
Sample 245	WB	Disc	Sanger	Beta+ Thal	Trp38X	5817	0.40	Het	Beta+ Thal, Sickle cell heterozygote
					Glu7Val	6023	0.48	Het	
Sample 462	WB	Disc	Sanger	Beta+ Thal	Gln40X	5934	0.47	Het	Beta+ Thal, Sickle cell heterozygote
					Glu7Val	5969	0.46	Het	
Sample 647	WB	Disc	Sanger	Glu7Val (Het)	Glu7Val	11,078	0.49	Het	Sickle cell heterozygote
Sample 693	WB	Disc	Sanger	Glu7Val (Het)	Glu7Val	5967	0.51	Het	Sickle cell heterozygote
Sample 926	WB	Disc	Sanger	Glu7Val (Hom)	Glu7Val	6700	0.99	Hom	Sickle cell disease
Sample 040	WB	Disc	Sanger	Beta^+^ Thal	Glu7Val	4847	0.45	Het	Sickle cell, Beta^+^ Thal heterozygote.
					Glu23X	4787	0.44	Het	
Sample 1077	WB	Disc	Sanger	Beta^+^ Thal	Glu 7Val	3741	0.46	Het	Sickle cell, Beta^+^ Thal heterozygote.
					Glu23X	3688	0.44	Het	
Sample 1221	WB	Disc	Sanger	Beta^+^ Thal	Glu7Val	3287	0.45	Het	Sickle cell, Beta^+^ Thal heterozygote.
					Glu23X	3258	0.45	Het	
Sample 832	WB	Disc	Sanger	Beta^+^ Thal	Glu7Val	3806	0.48	Het	Sickle cell heterozygote
Sample 750	WB	Disc	Sanger	Beta^+^ Thal	Glu7Val	3619	0.47	Het	Sickle cell, Beta^+^ Thal heterozygote.
					C.92 + 1G > T	2169		Het	
Sample 1181	WB	Disc	Sanger	Beta^+^ Thal	Glu7val	3111	0.46	Het	Sickle cell heterozygote

*WB* Whole blood, *DBS* Dried blood spot, *Val* Validation, *Disc* Discovery, *IEF* Isoelectric focussing, *Het* Heterozygous mutation, *Hom* Homozygous mutation, *VAF* Variant allele frequency

### Dried blood spot collection

The DBS samples were collected from newborn heel pricks using standard lancets. DBS collection, transportation, and storage were performed according to the manufacturer’s protocols (Lasec Diagnostics, Ndabeni, South Africa) and NBS guidelines from the Clinical and Laboratory Standards Institute [18].

### Whole blood collection

Four millilitres of whole blood were collected from each participant into an ethylenediaminetetraacetic acid (EDTA) tube at the Haematology Clinical Research Laboratory (HCRL) by venepuncture, according to HCRL Blood collection SOP version 01. The samples were tested for full blood picture (FBP) by Sysmex XT 2000i haematology analyser and haemoglobin subtyping and quantification by Bio-rad variant NBS HPLC. Also, the buffy coat was isolated by centrifugation of samples at 3000 rpm for 10 min and stored at -80 °C until use.

### SCD screening

Screening for SCD was performed at MUHAS HCRL: 3.2 mm of the DBS was punched by an automated DBS puncher (Wallac DBS Puncher) from PerkinElmer, Massachusetts, USA followed by IEF analysis, conducted according to the manufacturer’s protocol (Wallac RESOLVE Haemoglobin System). Samples showing abnormal results such as those found with the sickle gene (FAS), those found to be positive for SCD (FS) and those found to have unidentified variants FAV for the first test were re-punched and retested by HPLC. Results were interpreted by a trained laboratory technician and confirmed by the laboratory manager. Confirmed results were entered into the program’s database using the unique sample identification number.

### Molecular testing using the ONT Nanopore MinION

DNA was extracted from two 3.2 mm DBS punches or whole blood using QIAamp DNA Blood Min kit (Qiagen, Hilden, Germany) and the process took 1 h and 30 min to complete extraction. All DNA samples were quantified using either a Qubit high-sensitivity assay for DBS samples or a broad range assay for whole blood (Thermo Fisher Scientific, Waltham, MA, USA) and the process took 5 min to complete quantification. The β-globin gene locus was enriched by an in-house method developed at Oxford University, and each sample was given a unique barcode. Samples were quantified by Qubit broad range assay (Thermo Fisher Scientific, Waltham, MA, USA) and pooled in groups of 8–10 in a final concentration of 1 μg. Sequencing tethers and adaptors were ligated and the library was prepared for sequencing according to the SQK-LSK109 protocol (Oxford Nanopore Technologies, Oxford, UK) (Fig. 1b). Sequencing was performed using a FLO-MIN107 flowcell on a MinION mk1b instrument (Oxford Nanopore Technologies, Oxford, UK). Raw sequencing reads were analysed using a bespoke bio-informatics analysis pipeline (Fig. 1b). All variants called were subjected to additional quality filters (minimum depth of coverage: 200, Phred quality score:15. A VAF cut-off of 40–60% for heterozygous or > 75% for homozygous was applied, based on the range of VAFs of reference single nucleotide polymorphisms across the beta-globin locus.

*The methodology is subject to a licensing agreement. More detailed information is available on request.*

## Results

### Validation cohorts

Sequencing on a MinION instrument for 2 h generated 0.4Gb of sequence data across the 6 individuals of the whole-blood validation cohort, with an average depth of 36,000x over the *HBB* locus and an average quality score of Q20. Genotypes were classified as either heterozygous or homozygous based on their variant allele frequency (VAF) values (40–60% for heterozygous, ≥75% for homozygous). There were 13 germline variants in our WB validation cohort. Of these, 7 were synonymous changes, 2 were non-synonymous and 4 were found to affect the *HBB* initiation codon. All 6 individuals contained a homozygous non-pathogenic same-sense polymorphism (average VAF 99.7%, range 99.4–99.9) affecting the His3 residue (HBB; c.T9C). 2 individuals were heterozygous for the pathogenic HBB: c.20A > T mutation associated with SCD, with VAFs of 46 and 50%. 4 individuals were found to have HBB: c.2 T > C mutations, resulting in a loss of the *HBB* initiation codon. All 4 individuals were part of a single-family group, 2 parents and 2 daughters. Mother, father and 1 daughter were heterozygous for the variant (VAF 56, 44 and 41% respectively), while the remaining daughter was homozygous, with a VAF of 77%. All variants described were also identified by Sanger sequencing (Tables 1 and 2).

The 9 newborn DBS samples were sequenced in two groups, for 2 h each, and generated a total of 0.7Gb of sequence data, with an average depth of coverage of 48,000x and an average quality score of Q20. Of the 18 mutations identified, 9 were synonymous and 9 were nonsynonymous. All synonymous mutations were His3His variants, including 1 heterozygous (VAF 55%) and 8 homozygous (average VAF 99.3%, range 97.9–99.9%). The SCD-associated HBB; c20A > T variant was found in all 9 individuals, 5 heterozygous (average VAF 56.6%, range 44.7–72.6%) and 4 homozygous (average VAF 88.6%, range 80.7–95.1%). These variant calls were consistent with previously obtained data from IEF screening (Tables 1 and 2).

Overall, there was 100% concordance for identifying the SCD status between standard tests and Nanopore sequencing. In addition, the Nanopore sequencing approach detected additional variants of clinical significance in the beta-globin locus. There were no sequencing failures that required repeat analysis.

### Discovery cohort

We then extended our analysis to a discovery cohort of 18 patients, using DNA extracted from both whole blood (*n* = 12) and dried blood spots (*n* = 6). Across three sequencing runs, lasting 2 to 4 h, we generated 1Gb of sequence data, with an average quality score of Q20, and an average depth of coverage of 17,599x. There were 44 variants (22 synonymous, 14 nonsynonymous, 6 stop-gains and 2 splice-site) across all 18 individuals (Table 2). Again, all samples contained the common His3His polymorphism, while sample 245 harboured an additional synonymous polymorphism affecting the Lysine residue at position 60 (HBB; c.180G > A). The HBB; c 20A > T mutation was identified in 12 individuals, 1 homozygous and 11 heterozygous, with average VAFs of 94 and 47%, respectively. In 2 patients we identified a heterozygous variant located in the splice-donor region of exon one (HBB; c.92 + 1G > A) and (HBB; c.92 + 1G > T), also referred to as IVS1-1G > A and IVS1-1G > T. Stop-gain mutations were found in two individuals, both located in exon 2 (HBB; c.114G > A and HBB; c.118C > T), predicted to result in the loss of ~ 75% of the coding region (Table 2).

**Fig. 2:**
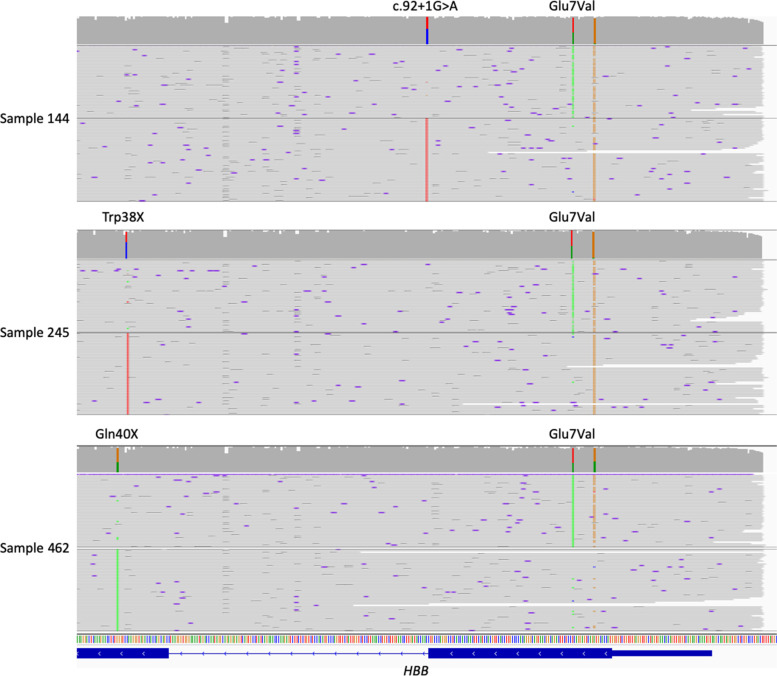
Mutations in the β-globin gene can be accurately phased across multiple exons due to long-read nanopore sequencing. Top: The c.92 + 1G > A variant in sample 144 is phased with the wild-type allele at the Glu7 residue. Centre and bottom: In both samples 245 and 462, the stop-gain variant is in phase with the wild-type allele at Glu7

As samples 144, 245 and 462 were all also heterozygous for the HBB; p. Glu7Val variant, we used WhatsHap to determine the phasing for each mutation pair. In all three cases we were able to demonstrate that the reference allele at the Glu7 residue is *in trans* with the alternate allele for the second mutation (HBB; c.92 + 1G > A, HBB; c.114G > A and HBB; c.118C > T, respectively, Fig. 2). This information is expected to have a significant impact on the phenotype of the patient, and would be missed by a protein-based SCD screening test.

We found no mutation in 6 dB samples that were excluded in Table 2 that contain only non-synonymous mutations.

### Cost analysis

One of the aims of this study was to develop a low-cost assay to screen for sickle cell and β-thalassaemia mutations, which would enable the assay to be adopted in low and middle-income countries (LMIC). As a consequence of amplifying the full β-globin locus in each patient, we were able to incorporate molecular barcodes for each patient during the library preparation stage. This allowed us to sequence multiple samples in a single run, using their barcode sequences to differentiate between them during data analysis. One advantage of the Oxford Nanopore flow cell is that they can be flushed without carry-over, allowing for a second sequencing run, on the same flow cell. On this basis, we prepared 24 samples per library, sequencing 8 libraries per flow cell. Thanks to this approach, we estimate the current cost of the test to be £11.57 per sample for consumables (Table 3). While this does not include hands-on time for library and bioinformatics analysis, the consumable cost compares very favourably with that of other sequencing methods and even with those of IEF and HPLC.

**Table 3:** Cost analysis of sickle cell and β-thal screening by nanopore sequencing. Cost estimates are based on 24 samples per library, and 8 libraries sequenced per flowcell

Item	Reactions per item	Cost per reaction	No. required per sample	Cost/test
QiAmp DNA blood mini kit	250	£1.86	1	£1.86
Enrichment Step 1	200	£0.72	1	£0.72
Enrichment Step 2	1000	£0.93	2	£1.86
Enrichment Step 3	50	£9.24	0.04	£0.41
PCR barcoding kit	960	£1.42	1	£1.42
Ligation sequencing kit	6	£80.00	0.04	£3.33
SpotON flowcell	192	£1.98	1	£1.98
**£11.57**				

*ONT* Oxford Nanopore Technologies

## Discussion

Increasingly, long-read sequencing methods, in particular Nanopore sequencing, are being used to address questions in clinical research, including whole-genome sequencing [19], targeted sequencing in cancer [20, 21], and genes with complex genomic structure, such as *GBA* [22]. The advantages of the Nanopore platform over more established techniques, in particular the short turn-around time and low cost of entry, make it an attractive proposition to labs running with limited funds. Our approach allows for the interrogation of different types of mutations across the entire beta-globin locus causing SCD or thalassaemia, including point mutations, stop gains, and deletions.

We show that Nanopore sequencing confirms heterozygous and homozygous haemoglobin variants, including identification of 100% of carriers of sickle cell variants. Moreover, we identify a family with the IVS1-1G > A mutation that has been shown to inactivate the IVS1 5′ splice site: as a consequence, three nearby cryptic splice sites are used [23], resulting in incorrectly spliced RNA, and a β^0^-thal phenotype. Little is known about the prevalence of thalassaemia in Sub-Saharan Africa and the underlying genetic mutations. The IVS1G > A mutation is found in patients of Arabic descent, and it is, therefore, plausible that it also occurs along the coastal region of East Africa. Our methodology would be ideally suited to study the prevalence of β-thalassaemia in the region.

In sub-Saharan Africa, there are increasing efforts to screen and diagnose genetic diseases at birth from DBS. These include SCD and thalassaemia. These efforts need to be complemented by accurate, simple, reliable, and affordable testing which will be used to diagnose multiple conditions. In this study we tested the use of DNA from both whole blood and DBS, the latter being the preferred method that may ensure inclusion of samples from remote locations and better fits the newborn screening algorithm. From our findings, DNA extracted from DBS generated MinION sequencing data of equivalent quality to whole-blood derived DNA.

This enabled us to address the big challenge of inconclusive results in NBS caused by incomplete Hb switching or co-inheritance of other haemoglobin variants. For example, we identified an individual with a heterozygous IVS1-1G > A variant *in trans* with a heterozygous Glu7Val mutation, as a result of using a long-read sequencing approach.

Although it is known that accurate diagnosis of genetic conditions is achieved through genotyping or sequencing, the required investment, cost, and expertise for conducting sequencing and data analysis have been challenging to put in place. The developed assay has proven to be feasible due to the minimum up-front investment required and low running cost. Also, with minimum hands-on wet lab training from the Oxford Molecular Diagnostic Centre (OMDC), local laboratory personnel at MUHAS can perform the assay. Most importantly, the technology does not require procurement and maintenance of expensive capital equipment and, although bioinformatics data analysis will require further rigorous standardisation, it can be securely performed in the cloud. This project has served as a platform for training by providing relevant sequencing data for trainees in bio-informatics. The training was delivered online and included the use of cloud servers, introduction into analysis tools and clinical variant identification and interpretation. Our long-term financial sustainability plan is based on a local social enterprise spin-out that delivers sequencing services to aid diagnostics and research.

## Conclusion

In a population such as Tanzania with a potential high prevalence of SCD coupled with other haemoglobin variants, DNA analysis allowing resolution of uncertain cases and confirmation of all positive cases is mandatory before pre-marital counselling and initiation of therapy. We show that single-molecule long-read sequencing, such as that offered by the MinION platform (Oxford Nanopore Technologies, Oxford, UK) enables the identification of SCD and other haemoglobin variants as well as the resolution of complex genetics that cannot be readily identified by HPLC and IEF. Its ability to sequence DNA from DBS and whole blood rapidly, at low initial cost, and without the need for initial capital investment make it an attractive DNA sequencing device to support haemoglobin research and diagnosis in low-middle-income countries such as Tanzania.

## Data Availability

The data supporting the findings of this study are available within the article. Raw data that support the findings of this study are available from the corresponding author upon a reasonable request.

## Abbreviations

DBS: Dried Blood Spot
DNA: Deoxyribonucleic acid
HPLC: High-Performance Liquid Chromatography
IEF: Isoelectric focusing
MNH: Muhimbili National Hospital
MUHAS: Muhimbili University of Health and Allied Sciences
NBS: Newborn screening
POC: Point of Care
VAF: Variant allele frequency
WB: Whole Blood
